# News Coverage of Face Masks in Australia During the Early COVID-19 Pandemic: Topic Modeling Study

**DOI:** 10.2196/43011

**Published:** 2023-08-16

**Authors:** Pritam Dasgupta, Janaki Amin, Cecile Paris, C Raina MacIntyre

**Affiliations:** 1 Department of Health Sciences Faculty of Medicine, Health and Human Sciences Macquarie University Sydney, New South Wales Australia; 2 Commonwealth Scientific and Industrial Research Organisation Data61 Sydney, New South Wales Australia; 3 Biosecurity Program, Kirby Institute University of New South Wales Sydney, New South Wales Australia

**Keywords:** face masks, mask, COVID-19, web-based news, community sentiment, topic modeling, latent Dirichlet allocation

## Abstract

**Background:**

During the COVID-19 pandemic, web-based media coverage of preventative strategies proliferated substantially. News media was constantly informing people about changes in public health policy and practices such as mask-wearing. Hence, exploring news media content on face mask use is useful to analyze dominant topics and their trends.

**Objective:**

The aim of the study was to examine news related to face masks as well as to identify related topics and temporal trends in Australian web-based news media during the early COVID-19 pandemic period.

**Methods:**

Following data collection from the Google News platform, a trend analysis on the mask-related news titles from Australian news publishers was conducted. Then, a latent Dirichlet allocation topic modeling algorithm was applied along with evaluation matrices (quantitative and qualitative measures). Afterward, topic trends were developed and analyzed in the context of mask use during the pandemic.

**Results:**

A total of 2345 face mask–related eligible news titles were collected from January 25, 2020, to January 25, 2021. Mask-related news showed an increasing trend corresponding to increasing COVID-19 cases in Australia. The best-fitted latent Dirichlet allocation model discovered 8 different topics with a coherence score of 0.66 and a perplexity measure of –11.29. The major topics were T1 (mask-related international affairs), T2 (introducing mask mandate in places such as Melbourne and Sydney), and T4 (antimask sentiment). Topic trends revealed that T2 was the most frequent topic in January 2021 (77 news titles), corresponding to the mandatory mask-wearing policy in Sydney.

**Conclusions:**

This study demonstrated that Australian news media reflected a wide range of community concerns about face masks, peaking as COVID-19 incidence increased. Harnessing the news media platforms for understanding the media agenda and community concerns may assist in effective health communication during a pandemic response.

## Introduction

In response to the initial phase of the COVID-19 pandemic, Australia implemented widespread testing, strict lockdown, and hotel quarantine measures [1,2]. In June 2020, most of the measures were relaxed and accompanied by less disruptive measures, such as increased testing capacity and contact tracing [2]. Along with the timely policy recommendations, the Australian community participated in health-protective behaviors, at least in the earlier stage of the pandemic [3]. A survey over the internet among Australian residents identified the determinants of their level of engagement with health-protective behaviors. Higher risk perception and following media coverage about COVID-19 were the predictors of greater engagement with protective behaviors [3]. Despite changing mask policy of international organizations, most of the respondents (79.7%) identified the policy recommendation at that time (face masks were only for sick people) [3]. At the same time, the respondents also reported that the mainstream news media was the most popular source of information in Australia [3,4] and they consumed more of the news on the internet than the offline version [4]. Overall, timely policy adoption, health communication, and widespread community adherence contributed to the early success of controlling the pandemic in Australia.

The SARS-CoV-2 is the viral pathogen that caused the COVID-19 pandemic in 2020 [5]. Face masks appear to be an effective tool in mitigating community transmission of SARS-CoV-2 [6]. While providing source control by trapping the infectious droplets and aerosols from an infected wearer, face masks also provide outward protection that reduces the viral inoculum from the environment to well-wearers [5]. This is particularly important for SARS-CoV-2 transmission, which may occur from symptomatic, minimally symptomatic, presymptomatic, and even asymptomatic people [5]. Evidence suggests that approximately 40%-45% of cases of COVID-19 are asymptomatic, which can silently spread the virus throughout the community [7]. In a systematic review and meta-analysis, funded by World Health Organization (WHO), researchers synthesized 172 observational studies related to physical distancing, face masks, and eye protection to prevent the transmission of COVID-19 in both health care and community settings [8]. The findings suggested that mask-wearing can reduce COVID-19 transmission by up to 85% and is often better with higher-end masks such as N95 and similar respirators [8].

In line with existing supportive evidence on community use of face masks, many countries that historically do not have a mask-wearing culture have initially adopted a mask-wearing policy, while other countries with an established mask-wearing culture have continued to use masks [9]. Australia did not mandate mask-wearing until community outbreaks of COVID-19 [10]. In response to a local outbreak of COVID-19, the State Government of Victoria introduced social distancing, a stay-at-home order, and a mask mandate in metropolitan Melbourne and the Mitchell Shire from July 22, 2020 [11]. A recent study showed that the mandatory mask-wearing policy increased community adherence to face masks and significantly reduced the number of COVID-19 cases during that outbreak [12].

During national crises and health emergencies, news media plays a central role in a contested landscape of uncertainty [13-15]. News media can communicate risks and preventive measures, and can shape public perceptions but may also contribute to disinformation. News stories during a health crisis often include public debate and policy responses to conflicting priorities [13,15]. In a complex situation such as a pandemic, news media allows health professionals, policymakers, and the general public to interact and exchange information [13,16]. During the early COVID-19 pandemic, Australian media covered a wide range of topics related to COVID-19 and consistently prioritized “mask-wearing” and “mental health” topics in their news stories. Health professionals and academic experts, along with political leaders, received coverage and therefore influenced the media agenda and were able to use media for advocating pandemic prevention strategies [17]. However, previous pandemics have shown that inadequate quantity and scientific value of the news content can also limit the effectiveness of public health policy responses [16]. In recent research, more than two-thirds of the respondents expressed concern about polarized media agenda, based on the sponsors and political ideology [18]. A higher media literacy can aid to distinguish factual news from fake news, advertisement, and poor journalism [18]. So, it is imperative to constantly scrutinize the news contents, particularly during a health crisis.

Agenda-setting theory has been considered one of the most popular conceptual frameworks in communication research [19], referring to the strong correlation between media coverage on certain issues and people’s perception of the importance of these issues [20]. Although the application of agenda-setting theory has been apparent in political contexts [21], the theory has long been applied in health issues [22] and crisis communication research [23]. In the context of COVID-19, agenda-setting became more diverse and interrelated where the media agenda was mostly shaped by the government and the public [24]. During the early stage of the pandemic in Australia, the national media coverage synchronized with the government-led agenda, including daily press conferences from State and Federal health leaders, and continuously relayed information from the health officials to the grassroots level [17].

While news media exercises the power of considerable discretion in choosing content for its storylines, media reporting on face masks can be designed within the skeleton of agenda-setting theory [20]. An ideal environment to shape public opinion and guide them toward informed decision-making mostly depends on the news reporting of mask-related agendas and ensuring their availability and repeated appearance on web-based news platforms. Based on the theoretical framework of agenda setting, the media is and has always been, a powerful tool of persuasion to facilitate desired health-protective behavior in the community [25]. Considering the importance of mask-wearing during the COVID-19 pandemic, it is useful to identify the media agenda and examine the news media contents, relevant topics, and their trends on face masks. It can provide useful insights into media narratives related to mask messaging.

At the beginning of the COVID-19 pandemic, Thomas et al [26] identified that a subtopic in Australian print media platforms was mask related. Basch et al [27] also reported similar findings in international news videos related to COVID-19 and found that only 6.2% of the video clips highlighted mask-wearing while caring for ill persons. Following the application and evaluation of a topic modeling algorithm in Chinese news data from the early COVID-19 pandemic, Liu et al [28] identified topics that emphasized mask use for both medical professionals and the general population. Yang et al [29] comparatively analyzed textual news media data and found that the China Daily newspaper paid more attention to mask-wearing throughout the study period than its counterparts from the United Kingdom and the United States. Moreover, multiple studies in this category analyzed news media contents from Iran [30], Brazil [31], and Italy [32]. Face masks appeared in these studies only as a subtopic under the broad topic of prevention and control measures, indicating a lack of media focus.

Lee et al [33] and Suh et al [34] attempted to investigate topics in mask-related news reports. In an earlier study, Suh et al [34] examined Korean news articles related to mask-wearing during 3 waves of COVID-19 in Korea from January 2020 to November 2020. By using a latent Dirichlet allocation (LDA)–based topic model, Suh et al [34] identified the major topics during the first and second waves of the COVID-19 pandemic in Korea. Additionally, Lee et al [33] applied a structural topic model algorithm on mask-related international news media data and identified underlying topics, and intertopic correlations.

Manual thematic analysis has limitations, especially in terms of scale. Natural language processing–based computational topic modeling techniques, in contrast, can handle complex and large amounts of data [35]. As part of the mixed method approach, topic modeling advances further qualitative interpretations and a deeper understanding of the topics [36].

The aim of the study was to examine news related to face masks as well as to identify related topics and temporal trends in Australian web-based news media during the early COVID-19 pandemic period.

## Methods

### Data Sources and Data Collection

Relevant data for the analysis were collected, both retrospectively and prospectively, from keyword-specific Google News search and Google News alert. Retrospective data were collected from January 25, 2020, to September 11, 2020, via Google News search. Subsequently, a customized Google News alert was set to collect prospective data from September 12, 2020, to January 25, 2021. For the retrospective and prospective data extraction, the keyword “mask” was used in conjunction with the ~ (tilde) symbol. That means the generated results, for both the Google News search and Google News alerts, contained the keyword or the synonyms (or both).

The results were then joined to form a combined data repository and named as “News topic data set.” In that repository, news titles containing the search keyword and its synonyms were regarded as most relevant, and therefore only those news titles were selected for the analysis. Additional eligibility criteria were established to include consistent, reliable, and uniform data.

### Selection Criteria

#### Inclusion Criteria

The inclusion criteria were (1) news titles in the English language and (2) news published by Australian news publishers only.

#### Exclusion Criteria

The study excluded irrelevant “mask”-related news articles such as beauty mask and sheet mask.

### Face Mask–Related News Trend

As part of the descriptive data analysis, the trend in the number of mask-related items published in news on the internet was explored by plotting the frequency of occurrence in relation to key events during the COVID-19 pandemic, face mask–related incidents, and policy announcements in Australia (as described in the Result section). The key events were obtained from the Australian Government’s website and national news reports.

### Exploratory Data Analysis by Topic Modeling

#### Overview

Exploratory data analysis of the news titles in the “News topic data set” was done by topic modeling technique. Topic modeling is a probabilistic computational method that identifies latent semantic patterns of word co-occurrence, also known as topic, in a collection of digital documents [37]. There are different topic modeling algorithms, particularly for short-text data such as news titles. They can be unsupervised, supervised, or semisupervised [38]. There are 5 frequently used topic modeling methods: LDA, latent semantic analysis, nonnegative matrix factorization, principal component analysis, and random projection [38]. LDA has the advantage of being an unsupervised model and some also consider the topics from LDA as easily interpretable. In a study that compared the various topic modeling algorithms, both LDA and nonnegative matrix factorization outperformed other methods and detected meaningful topics in 2 different textual data sets [38]. LDA is the mostly applied topic-modeling algorithm for textual data [39]. We used the LDA model as described by Blei et al [39] in 2003.

#### Data Preprocessing

**Figure 1:**
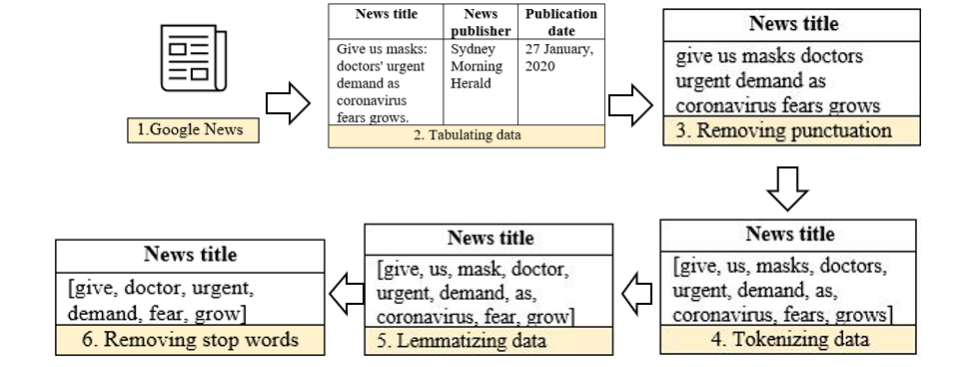
Steps of data preprocessing for latent Dirichlet allocation topic modeling.

Preprocessing of the news titles was performed in Python (version 3; Python software foundation) [40]. The data processing is illustrated with examples in Figure 1.

#### Vectorization

Meaningful numerical representation of the features in the corpus is called vectorization. It represents the textual data for better understanding by the machine and is often considered to be imperative in natural language processing applications [41]. Term frequency–inverse document frequency is the commonly used method for vectorization of textual corpus [41] and was hence used in this study.

#### LDA Topic Model Algorithm

We again used Python to develop the LDA model [40]. LDA requires the number of topics (k) to be predetermined by the researchers.

### Evaluation Matrices of LDA Models

#### Overview

As there is no statistical test to determine the number of topics (k), evaluation matrices can be used as a guide to identifying the best fit for the data set. We developed our model with different numbers of topics (k=1 to 20) and evaluated them according to both quantitative and qualitative matrices. Initially, we selected the top 3 models according to their performances on the quantitative evaluation scale. We then qualitatively analyzed those top 3 models and selected the best model for our data set.

#### Quantitative Matrices

Two quantitative matrices were used to evaluate the models.

#### Perplexity Measure

Perplexity is a statistical measure to estimate the performance of a probability model in predicting a sample. A lower perplexity score indicates a better model for predicting the existing topics in the data set [39]. However, sometimes perplexity does not correlate with human interpretability, as a result, additional measures are required to determine the best-fitted model.

#### Coherence Score

The coherence score refers to the degree of semantic similarity among high-scoring words in a topic [42,43]. It is proportionately related to the quality of the topics and their human interpretability [44]. A higher coherence score indicates a better-performing model [42]. The coherence score is considered the best quantitative evaluation matrix for topic modeling. So, we used it to evaluate our LDA topic models.

Both the perplexity measures and the coherence scores for our LDA topic models (with different numbers of topics) are shown in the *Results* section.

#### Qualitative Matrices

##### Interactive Topic Visualization

To visualize the topics, we used LDAvis [45], a bubble chart that presents the topics in a 2D space created by PC1 and PC2 (principal components 1 and 2). Each bubble represents 1 topic with the volume proportional to the frequency of the topic in the corpus. A well-designed topic model is indicated by nonoverlapping bubbles spread throughout the 2D plane. To identify the best-fitted LDA model, we compared the Intertopic distance maps of the top 3 models with their unique value of k.

##### Word Cloud

A word cloud for each topic shows dominating terms for the topic. The word cloud is constructed using the topic terms and their probabilities in the topic construction. Dominating terms can be easily identified and compared contextually, both within and outside the topic. The word cloud of our final LDA model is visualized in the Results section.

##### Topic in Context of News Titles

For a contextual analysis of the topics, we tabulated the most-representative news title for each topic and the proportion of the topic in that particular news title.

### Temporal Topic Trends

Upon selection of the best-fitted model, the model can be examined at both macro and micro levels. At the macro level, the model provides an overview of the individual topic throughout the observation period. In contrast, microlevel observation can use the temporal variation of the data and illustrate the trends of the topics. In this study, the observation period was separated per month, resulting in 13 time slices, spanning January 25, 2020, to January 25, 2021. Each news title was tagged with its dominant topic (calculated from the corresponding probability distribution). Then, the distribution of the dominant topics for news titles was plotted for the 13 time slices. The evolution of the topics is illustrated in a heat map.

## Results

### Overview

A total of 2404 mask-related Google News titles were identified in Australian web-based news websites from January 25, 2020, through to January 25, 2021. After excluding 59 irrelevant items (2.45%), a data set of 2345 mask-related news titles was finalized. The leading publishers were the national mainstream news media, such as ABC News, 7 News, and Sydney Morning Herald.

### News Trend Analysis

**Figure 2:**
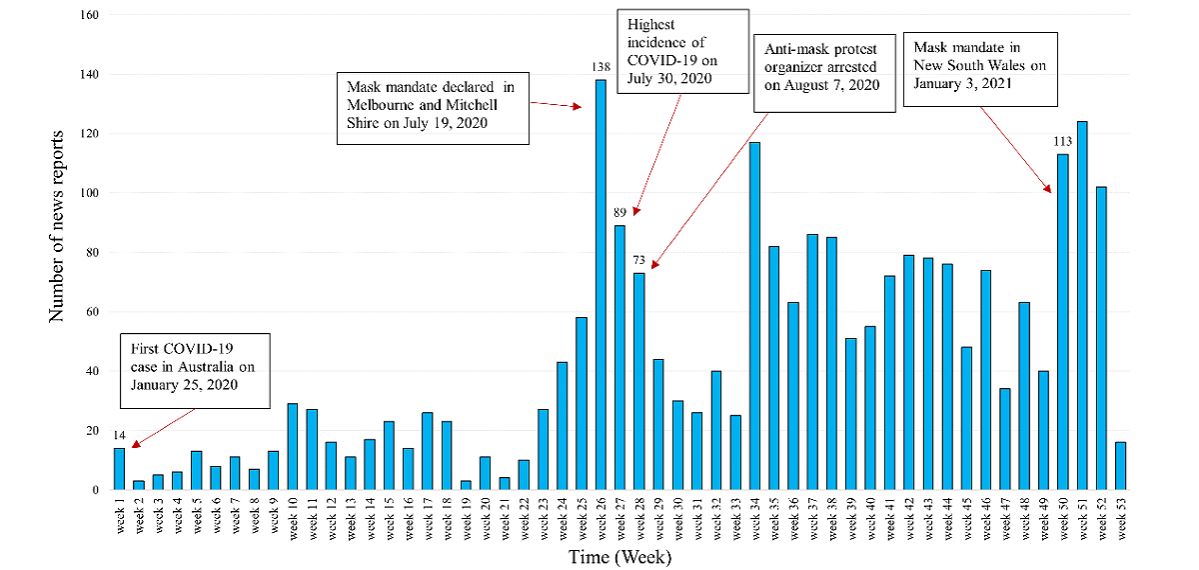
Weekly number of mask-related web-based news articles in Australia, 2020-2021 (Week 1 commences January 25, 2020).

Figure 2 presents the time trend of weekly published mask-related web-based news articles on Australian news websites for the duration of 2020-2021. The highest number (138 news articles) of news items (per week) was recorded on the 26th week (July 18 to 24, 2020). It corresponds to a pandemic wave in Melbourne, Victoria, and a mask mandate introduced in Melbourne and Mitchell shires on July 19, 2020.

### Evaluation of the LDA Topic Model

#### Quantitative Evaluation

**Figure 3:**
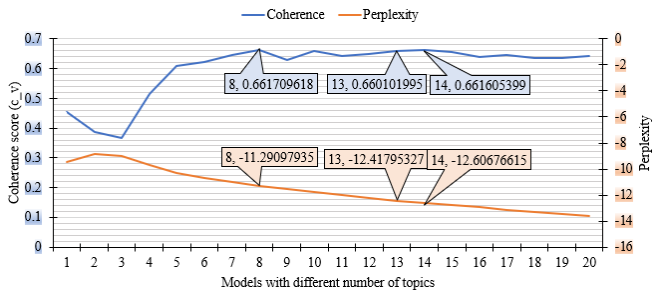
Perplexity measures and coherence scores of 20 topic models.

Figure 3 contains line graphs for the coherence scores and perplexity measures of LDA models with 20 different numbers of topics. Based on the quantitative evaluation matrices, the selected models were the model with 8 topics, 13 topics, and 14 topics.

#### Qualitative Evaluation

**Figure 4:**
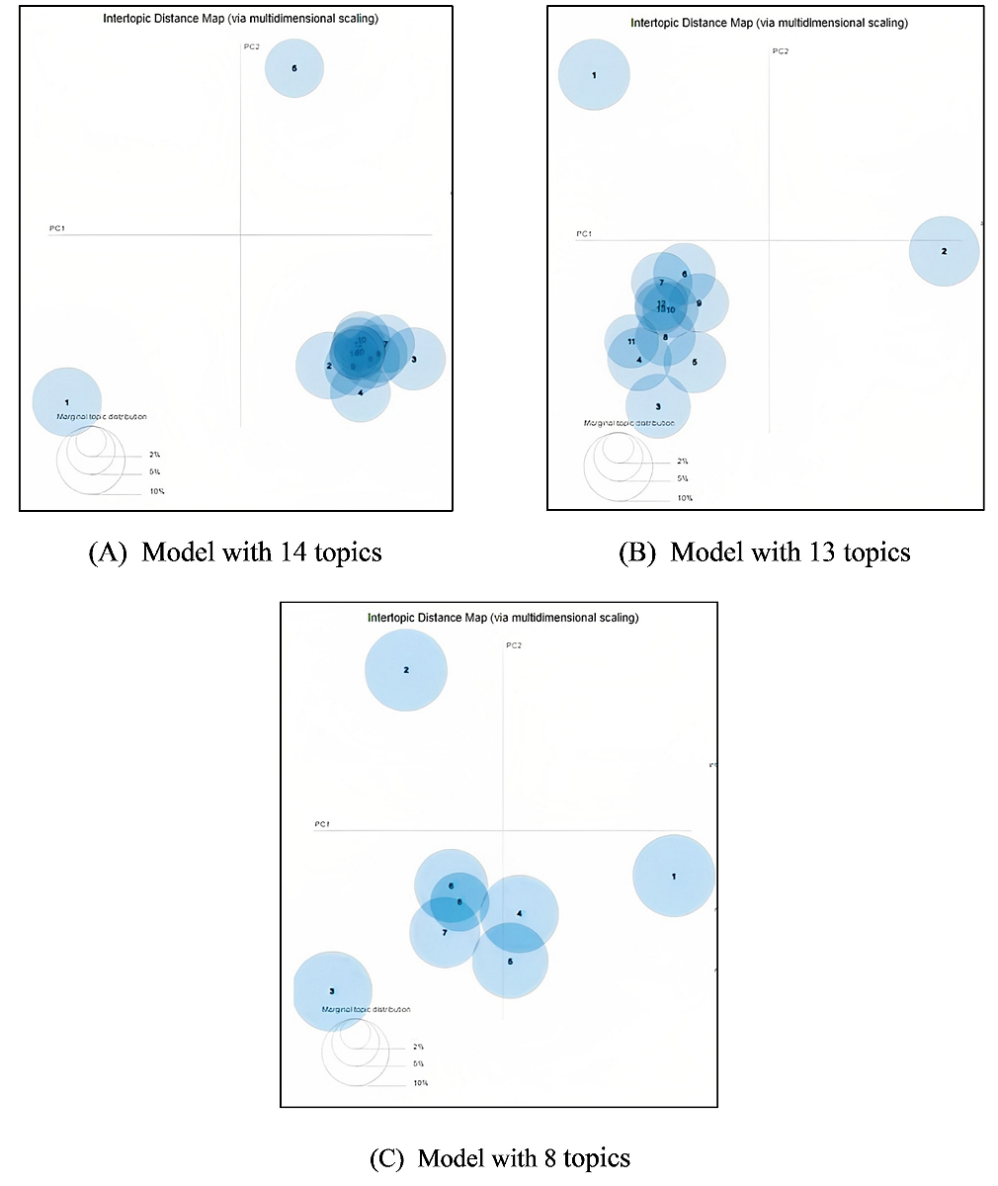
Intertopic distance maps of topic models. For a higher-resolution version of this figure, see Multimedia Appendix 1.

As part of the qualitative evaluation, we used the Intertopic distance maps of the models. Figure 4 visualizes the Intertopic distance maps for topic models with 14, 13, and 8 topics. Overall, the model with 8 topics shows less overlap and more evenly distributed topics. This is the model we selected as the best-fitted model for our “News topic data set.”

### Interpreting the Resulting Topics

Multimedia Appendix 2 shows a visualization of the topics obtained, using word clouds. As already mentioned, the size of the topic terms depends on their probabilities in the topic. The figure also includes the proportion of overall tokens for each topic, whereas the term “token” represents an individual word in the corpus. The bigger the proportion of the tokens for each topic, the better it can be interpreted.

We named the topics according to their dominant terms: mask-related international affairs (T1), introducing mask mandate in places such as Victoria and Sydney (T2), implementing mask mandate in Sydney (T3), antimask sentiment (T4), miscellaneous topic (T5), mask use in public places (T6), promoting mask-wearing (T7), and mask-wearing after the lifting of restriction (T8).

The miscellaneous topic (T5) included news articles related to the challenges of mask-wearing in western culture. In addition to the example in Table 1, another news title under this topic is, “Video shows GOP Sen. Dan Sullivan and Democratic Sen. Sherrod Brown arguing after the Republican refused to wear a mask while speaking in the Senate.” T5 also identified face masks as a canvas for individual and community expression on local and global issues. A few examples of news titles are, “Twins Max Kepler sorry for Blue Lives Matter mask amid Minneapolis protests” and “Ai Weiwei masks flip the bird, a kebab knit and gorgons: corona art.” Table 1 contains a table of the topics, their keywords, and most representative news titles with the probability of that given topic in that particular news title.

**Table 1:** The most representative news of 8 topics.

Topic number	Topic keywords	Most representative news title	Topic contribution in the title, %
1	restriction, international, eased, biden, melbourne, lifted, shopper, dont, open, one	Melania Trump votes without a mask on US election day as Donald Trump, Joe Biden, and Kamala Harris make last-minute pitches.	65.58
2	new, mandatory, fine, victoria, greater, premier, sydney, 2021, victorian, officer	Bow Wow performed in a packed Houston nightclub for scores of mask-less attendees—and people have a lot to say about it.	61.42
3	sydney, mandated, ease, must, resident, fined, strain, public, mandatory, could	NSW Health Minister Brad Hazzard apologizes for calling Labor leader Jodi McKay “quite stupid” in mask stoush.	63.99
4	mandate, charged, flight, police, supermarket, antimask, right, woman, barty, refusing	Delta has now banned some 350 passengers for refusing to wear masks during flights — and it is adding 100 people a month to its no-fly list.	65.51
5	man, 10, warning, set, best, required, latest, biting, alert, plan	HHS secretary Alex Azar says wearing masks is “a difficult message for all Western democracies.”	58.82
6	airport, brisbane, test, case, office, wa, opera, stock, get, around	Armidale’s Isabelle and Lillie Kelly go from homemade scrunchies and hair ties to face masks.	60.44
7	rule, smart, pushup, likely, confirms, made, without, shopping, half, day	Queen Elizabeth wears a face mask in public for ceremony to mark the centenary of burial of the Unknown Warrior.	60.24
8	nsw, masked, buy, across, issue, men, bali, tourist, mandate, order	Coronavirus in Ohio: Parents sue health department director Lance Himes over K-12 school mask mandate.	62.22

### Temporal Topic Trends

**Figure 5:**
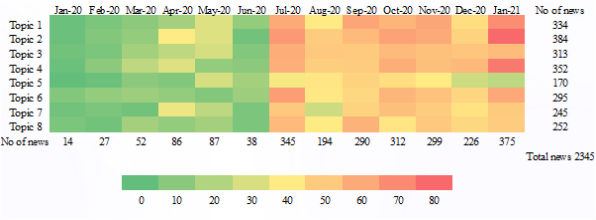
Heat map of the temporal distribution of mask-related topics.

Figure 5 is a heat map of the temporal trends of the topics identified in our model.

The heat map distinctly represents both similar and opposite trends of topic evolution. Few mask-related news items were published in the initial 6 months (January 2020 to June 2020), followed by a sharp increase afterward for all the topics. In the first half of our analysis, January 2020 to June 2020, news relating to T2, T4, and T7 appeared more frequently. Then T2 and T4 were predominant with a peak in January 2021 (77 items for T2 and 70 items for T4).

## Discussion

### Principal Findings

During the COVID-19 pandemic, national mainstream media organizations were at the forefront of reporting mask-related stories in Australia. We conducted a series of LDA analyses of our corpus on mask-related news items, using quantitative and qualitative measures to identify the best-fitted model (the best number of topics). Even though both coherence score and perplexity measure were used to quantitatively assess our LDA model performances, the utility of perplexity measure was deemed negligible in our LDA model selection. Based on the evaluation matrices, we identified 8 topics related to face masks, with 3 key topics: mask-related international affairs (T1), introducing mask mandate in places such as Victoria and Sydney (T2), and antimask sentiment (T4). Both T2 and T4 were dominant and peaked in January 2021. This study demonstrated the utility of a data-driven approach to analyzing news media and identified how it communicated health-related information during a public health crisis.

During the study period, there were 2 distinct waves of mask-related news publications in Australia. The second wave was much stronger than the first and was mostly caused by local spikes in COVID-19 cases and the introduction of mandatory mask-wearing in different states. In a comparative cluster analysis, Yang et al [29] found a similar trend in the United Kingdom and the United States: both countries initially experienced relatively few news items related to mask, followed by an increase in response to local COVID-19 outbreak. In contrast, Chinese news media consistently published mask-related news since the pandemic started [29].

Similar to our study methodology, Suh et al [34] used an LDA topic model for their Korean news data set on face masks. However, they only used a quantitative method to evaluate their model, and their semantic similarity score of topic keywords is smaller (0.52) than the one in our study (0.66). It means that the topics in our study are more clearly defined and more easily interpretable.

Topic wise, T1 (mask-related stories in international news) was one of the major topics identified in our corpus, with 334 news titles. The most representative news title under this topic showed that Australian web-based media coverage of mask-related international events was mostly from the US political arena. Considering the escalating conflicts between politicians and the later politicization of mask use in the US election, this topic reflects the centralized role of the United States in overall “mask madness” [46]. Lee et al [33] identified a similar topic in their study, which they labeled “President election.” On topic-trend analysis in our study, T1 constantly appeared from July 2020 onward. Following the recommendation of the US Centers for Disease Control and Prevention (CDC) on public masking, this topic covered an increasing number of news reports on public gatherings (protests, political campaigns) that might worsen the ongoing pandemic.

T2 of our LDA model was about introducing mask mandates, mainly in response to the new cases of COVID-19 in Victoria and Greater Sydney. This topic gained the highest media coverage, with a total of 384 news titles. On the temporal trend analysis, this topic reflected the government-led agenda on mask-wearing and evolved along with the mask-relevant policy changes across the states in Australia. The highest amount of T2 specific news coverage (77 items) was recorded during the mask mandate in the Greater Sydney region in January 2021, followed by the Melbourne mask mandate (56 items) in July 2020. The predominance of the second topic around the policy change implied that the reporting by Australian media was timely and in line with a policy maker’s guideline to promote mask-wearing [6]. Suh et al [34] identified a similar topic during the second outbreak of COVID-19 in Korea. Lee et al [33] also identified this topic in a large data set of international news about mask-wearing.

Our third topic (T3) was related to mask mandates in the Greater Sydney area, reflecting mostly actions taken by law-enforcement agencies. This topic also depicted different attitudes of the political leaders and public figures toward face masks and associated policy development. Over time, T3 was consistent from the time of the Melbourne outbreak in July 2020. Following the outbreak in the Greater Sydney region in January 2021, this topic exhibited an increased number of related news publications.

Besides T2, the next major topic was related to antimask sentiments (T4). In our study, a total of 352 news titles were recorded under this topic. Lee et al [33] had a similar finding in their analysis, concluding that the “Protesting movement” topic in mask-related news predominantly appeared after WHO’s pandemic announcement. This topic illustrates the media messaging on the conflict between the government agenda and public agenda in terms of mass masking strategy. The highest number of news items for this topic was related to the Greater Sydney mask mandate in January 2021 (70 news titles), followed by the Melbourne mask mandate in July 2020 (46 news titles).

With 295 relevant news articles, T6 was about news encouraging mask-wearing in public places and at events. Similarly, T8 described mask-related policy implementation while reopening offices and educational institutions. With 252 mask-related articles, this topic drew more attention during September 2020. Overall, T6 and T8 focused on the universal adoption of face masks and integrating the concept of mask-wearing in regular and recreational events. These topics suggest the importance of mask-wearing to minimize COVID-19 transmission while getting “back to normal.” In the United States, mask-wearing policies related to school-reopening strategies effectively reduced the community transmission of COVID-19 [47]. In international news media data, Lee et al [33] also identified multiple topics about mask-wearing in educational institutions, local businesses, sports, and family events.

The next topic in our study, T7, was about the innovative promotion of mask-wearing. With 245 news titles, this topic developed since the recommendation of the US CDC on wearing nonmedical face masks in public places. During the second phase of our study, from July 2020, T7 evolved constantly and might contribute to encouraging mask-wearing by framing positive social messages in the news content. Furthermore, these positive social messages can contribute to developing positive social cognitions in the community [48].

Last, T5 was regarded as a miscellaneous topic with the least news coverage. Topic-specific keywords did not cluster under any specific topic. On the temporal analysis, the number of such miscellaneous news gradually decreased, making a place for more structured and topic-specific media messaging in Australian web-based news platforms. In contrast, Suh et al [34] identified an increasing number of news items under miscellaneous topics during the third wave of COVID-19 in Korea.

Our study has several strengths that can be attributed to both the methodology and the outcome of the study. Methodological strengths include the inclusion of both national and local news media reports on face masks in Australia. Although international news articles rarely reflect the Australian national agenda, selective news websites may not represent all the national issues. Additionally, the data collection tool Google News is distinctly advantageous over other news databases such as LexisNexis. Google News provides more reliable data and can capture more local news and nonprint articles that are often overlooked in other database searches [49]. Moreover, Google News is a free data collection tool that permits a wide range of researchers to access and analyze news data, and therefore analyses are amenable to assess for replicability.

### Limitations

There are several limitations that can be attributed to this study. One of the major limitations is analyzing only the news titles. They might not provide adequate context to capture appropriate topics for corresponding news articles. However, considering the structural integrity of news titles in summarizing news themes, it is likely still meaningful. In future research, we could aim at capturing both the title and the snippet returned by the search engine. By using images from the news and their associated titles, visual topic modeling could also be a novel approach to address the limitations of only news title analysis [50]. Furthermore, there are certain limitations to Google News. It sometimes restricts access to news that requires a subscription. Methodologically, LDA may not necessarily perform well when analyzing short news titles. For example, the most representative news title for T2 does not represent the topic in our LDA model (see Table 1). Additionally, we did not perform a sentiment analysis on the news title. Such an analysis could be included in future work [51,52]. Finally, our study of news items could be complemented by an analysis of social media discourse.

### Conclusions

Appropriate preventive strategies, health communication, public education, and active community participation have always been at the center of effective pandemic responses. News media platforms, as primary sources of updated health information, could serve the interests of the government and the community by disseminating information about health policies and healthy behaviors. However, the media can also amplify dissent and uncertainty. This study highlighted the public health recommendations about mask wearing and diverse aspects of mask use (or not) in the media. Timely, targeted, and transformative media messages can rapidly spread around the world, and therefore those messages can facilitate local and global community adoption of mask-wearing which will benefit all.

## Appendix

Multimedia Appendix 1 Intertopic distance maps of topic models.

Multimedia Appendix 2 Word clouds of 8 topics.

## Abbreviations

CDC: Centers for Disease Control and Prevention
LDA: latent Dirichlet allocation
WHO: World Health Organization
