# Supplementary figures and images for: News Coverage of Face Masks in Australia During the Early COVID-19 Pandemic: Topic Modeling Study
Source: JMIR Infodemiology. 2023 Aug 16;3:e43011. doi: 10.2196/43011 (PMC10434701; doi:10.2196/43011)

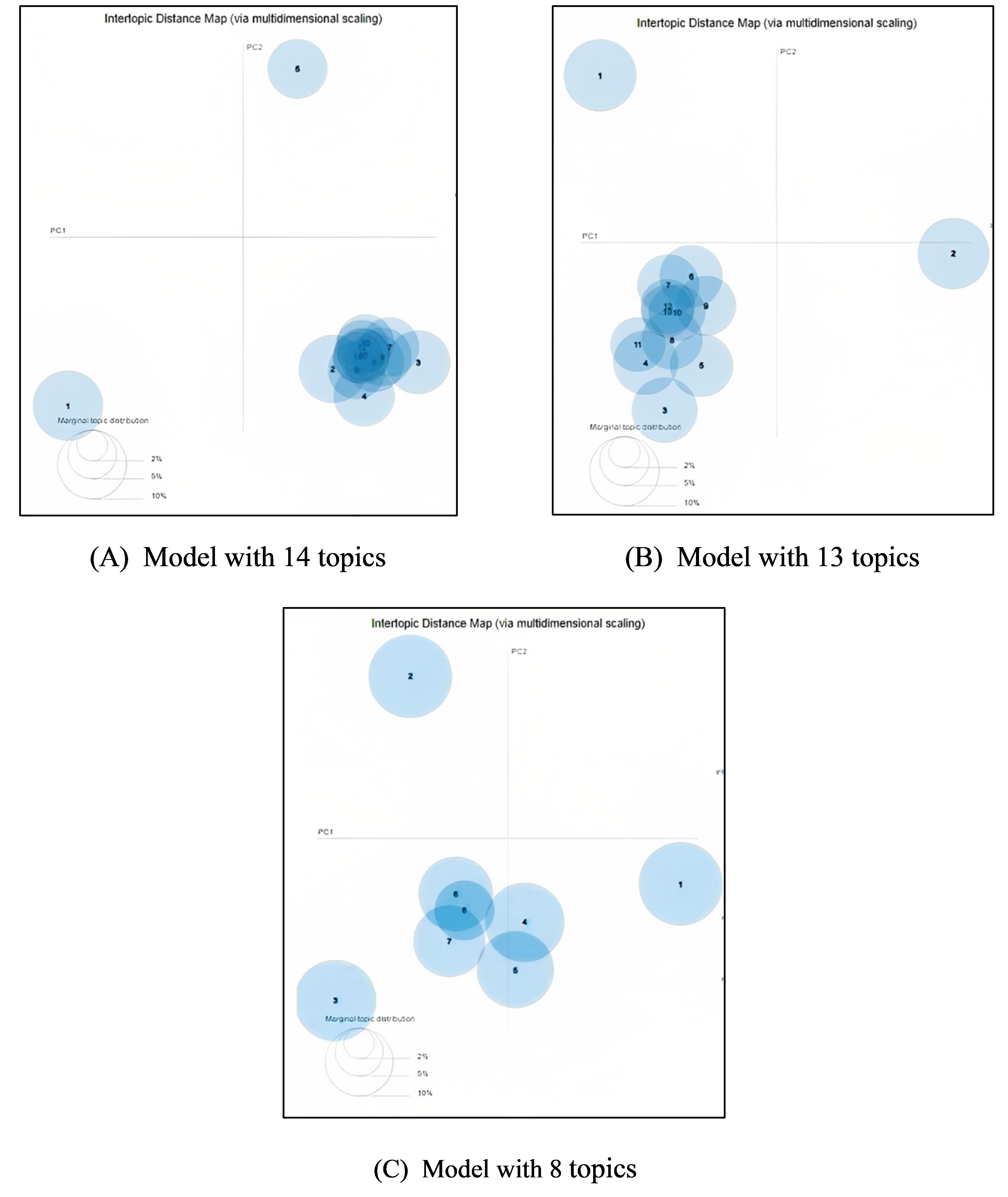

Supplement: Multimedia Appendix 1 [file infodemiology_v3i1e43011_app1.png]

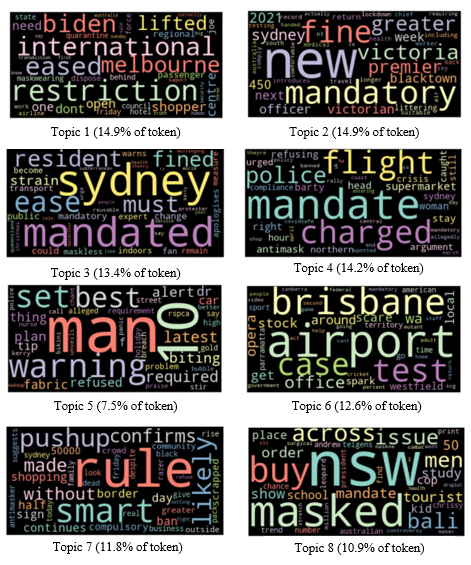

Supplement: Multimedia Appendix 2 [file infodemiology_v3i1e43011_app2.png]
